# Quantifying the Size-Dependent Shear Banding Behavior in High-Entropy Alloy-Based Nanolayered Glass

**DOI:** 10.3390/nano14060546

**Published:** 2024-03-20

**Authors:** Kaiqing Dai, Chun Zhang, Wenjun Lu, Jianjun Li

**Affiliations:** 1 College of Mechanical and Electrical Engineering, Central South University, Changsha 410083, China; 203701021@csu.edu.cn (K.D.);; 2 State Key Laboratory of Precision Manufacturing for Extreme Service Performance, Central South University, Changsha 410083, China; 3 Department of Mechanical and Energy Engineering, Southern University of Science and Technology, Shenzhen 518055, China

**Keywords:** nanolayered glass, nanoindentation, shear banding, amorphous/amorphous interface

## Abstract

Extensive research has shown that nanolayered structures are capable of suppressing the shear banding in metallic glass in nanoindentation experiments. However, the specific mode and mechanism of the shear banding underneath the indenter remains unknown. Also, the quantification of shear banding-induced strain localization is still a challenge. Herein, the size-dependent shear banding behavior of a CuTiZrNb high-entropy alloy-based nanolayered glass with individual layer thicknesses (*h*) ranging from 5 to 80 nm was systematically investigated by nanoindentation tests. It was found that the hardness of the designed structure was almost size-independent. Yet, a clear transition in the deformation modes from the cutting-like shear bands to the kinking-like ones was discovered as *h* decreased to 10 nm. Moreover, multiple secondary shear bands also appeared, in addition to the primary ones, in the sample with *h* = 10 nm. The transition leads to an obvious strain delocalization, as clearly illustrated by the proposed theoretical model, which is based on the assumption of a pure shear stress state to quantify the shear banding-induced strain localization. The strain delocalization results from the higher density of amorphous/amorphous interfaces that exhibit the change in morphology with a refined layered glass structure.

## 1. Introduction

Metallic glass (MG) materials (or amorphous solids) usually possess ultra-high strength and hardness, due to their unique atomic structures lacking long-range orders, which prevents the initiation and motion of dislocations, as compared with their crystalline counterparts [1,2,3,4,5,6]. However, the lack of dislocation activities is the source of their near-zero plasticity since single shear banding-dominated deformation always generates catastrophic failure after the applied stress reaches the elastic limit of the material [7,8,9,10]. Therefore, improving the plasticity and tensile ductility of MGs while simultaneously maintaining their ultra-high strength has become an important issue in the field of materials science in recent decades [11,12,13,14]. Significant efforts have been made to achieve the above goal, and several typical and effective strategies based on interface engineering have emerged, which have generated the high density of crystalline/amorphous (C/A) and amorphous/amorphous (A/A) interfacial structures at the nanoscale [15,16,17,18,19,20,21,22,23,24].

The first effective strategy is to engineer MG-based composites by introducing ductile crystalline phases [25,26,27,28,29,30,31,32,33,34,35]. A typical example is a nanolayered 35 nm Cu/5 nm amorphous CuZr composite fabricated by magnetron sputtering [17]. The tensile test showed that the nanolayered composite exhibited a high flow stress of around 1.1 GPa and a tensile elongation to failure as high as 13.8%. The high tensile plasticity was attributed to the suppressed shear banding instability, due to the high-capacity sinks resulting from the high density of C/A interfaces. Another typical example is the supra-nano C/A dual-phase magnesium alloy, in which 6 nm MgCu_2_ nanocrystals were fully surrounded by 2 nm-thick magnesium-enriched amorphous shells [36]. This special C/A core–shell supra-nano structure enables the strongest magnesium alloy film with a near-ideal strength of 3.3 GPa by blocking the propagation of localized shear bands (SBs) in the C/A interfaces and dividing and rotating the nanocrystalline grains. Similar supra-nano structures and superior mechanical properties have also been achieved in an aluminum alloy [37] and a CrCoNi-Fe-Si-B high-entropy alloy (HEA) [38].

Another effective strategy is to develop various amorphous alloys with high density of nanoscale A/A interfaces by taking advantage of the size-dependent plasticity of MGs [12,16,18,23,39,40,41,42]. A typical amorphous material with nanoscale A/A interfaces is nanoglass [16,18,39,40,43,44,45,46], the microstructure of which is similar to that of nano-grained metals. However, the grain interior and boundary of the latter are replaced by the same amorphous phases but with different elemental contents. Many experiments and atomic simulations have shown that the plasticity of nanoglass materials can be substantially improved due to the nanoscale A/A interfaces [15,16,23,39,40,41,46,47]. For example, Wang et al. [48] produced a Sc_75_Fe_25_ nanoglass by the inert-gas condensation technique, which possessed nano-grains with a diameter of 10 nm and glassy interfacial regions with a thickness of 1 nm. The in situ tensile test of the designed nanoglass exhibited a plastic strain as high as 15%, while the MG counterpart showed almost no plasticity. Nandam et al. [43] prepared a Cu_50_Zr_50_ nanoglass with a particle size of 6 nm by using magnetron sputtering in an inert-gas condensation system, the compositions in the grain interior and interfacial regions of which were determined to be Cu_45_Zr_55_ and Cu_57_Zr_43_. The shear banding behavior was tested by microindentation, which showed that the shear banding observed in the nanostructure-free sample disappeared in the nanoglass sample. The homogeneous deformation and the enhanced resistance to the shear banding-induced strain delocalization was attributed to the interfacial regions of high free volume, which promotes the nucleation of many shear transformation zones (STZs).

In addition to experimental studies, molecular dynamics simulations have also been conducted to investigate the underlying deformation mechanisms of nanoglass with high plasticity [23,41,47,49]. For instance, Adibi et al. [47] studied the grain size-dependent Cu_50_Zr_50_ nanoglass with grain sizes varying from 15 to 5 nm by molecular dynamic simulations. The simulations revealed a transition from a single shear banding with a grain size larger than 10 nm to a homogeneous superplastic flow deformation with a grain size smaller than 5 nm. This transition originated from the uniformly distributed STZs and the homogeneous elastic energy release with grain refining.

Since the size of the grains and the thickness of the interfacial phase of nanoglass are difficult to adjust in the experiments of inert-gas condensation, a bottom-up method has been used to accurately control the A/A composites. A widely used bottom-up technique is magnetron sputtering (physical vapor deposition), which alternatively stacks nanoscale amorphous layers of different materials or compositions to form the designed composites [50,51,52,53,54]. For example, Sharma et al. [44] prepared a Zr_55_Al_10_Cu_30_Ni_5_/La_55_Al_25_Ni_10_Cu_5_Co_5_ nanolayered glass (NLGs) with an individual layer thickness (*h*) of 200 nm by alternatively depositing the Zr-based and La-based amorphous targets in the magnetron sputtering system. It was found that the A/A layered interfaces completely suppressed shear banding in the amorphous alloy during nanoindentation tests, instead of the multiple SBs in the single-phase La-based or Zr-based MGs. The suppressed shear banding was believed to result from the blockage of SBs because of the existence of the A/A interfaces in the nanolayered structure. Recent experiments also showed that the shear banding behavior of nanolayered amorphous alloys was highly dependent on the layer thickness (i.e., the A/A interface spacing) [51,52,55]. For instance, Chen et al. [55] investigated the size effect of the nanoindentation behavior of Ni_60_Nb_40_/Zr_50.7_Cu_28_Ni_9_Al_12.3_ NLGs. Their results showed that the shear banding was gradually suppressed with the decrease of *h*, and that the shear banding fully disappeared in the samples with *h* ≤ 10 nm. This enhanced homogeneous deformation might result from more and wavier A/A interfaces.

The above extensive experimental investigations have shown that the A/A interfacial structures can enable significant plasticity in amorphous alloys without sacrificing strength, which is suffered by the MG-based composites with C/A interfaces due to the incorporation of the relatively softer metallic phases [56]. However, most of the existing studies have focused on the shear banding-induced surface morphologies during the indentation process. What happened inside the materials underneath the indenter remains unclear, especially the specific characteristics of the SBs, including the shear banding mode (cutting-like or kinking-like SBs) [21]. Another important issue is that a theoretical model that can be used to quantify the shear banding-induced strain localization in the amorphous alloys is still lacking.

Herein, in order to address the above critical issues, a systematic study was conducted to investigate the effect of layer thickness on the shear banding behavior of a HEA-based NLG (i.e., Cu*_x_*(TiZrNb)_100−*x*_ NLG) with a high density of nanoscale A/A interfaces by nanoindentation experiments. The nanoscale A/A interfacial structure is produced by alternatively co-sputtering Cu and TiZrNb targets under different sputtering powers for the two constituent layers. A combination of the detailed transmission electron microscopy (TEM) characterization of the indented areas and a theoretical analysis was employed. The results clearly show that the shear banding behavior is strongly dependent on the layer thickness, where the cutting-like shear banding is highly suppressed in the samples with refined layers. It was found that the decrease of layer thickness induces a transition of the shear banding mode, i.e., from the cutting-like SBs in the sample with *h* = 80 nm to a mixture of the cutting-like and kinking-like SBs in the sample with *h* = 40 nm, and finally to the kinking-like primary SBs accompanied by several secondary SBs in the sample with *h* = 10 nm. The theoretical analysis demonstrates that strain delocalization is enhanced with decreasing layer thickness in the Cu*_x_*(TiZrNb)_100−*x*_ NLGs due to more and wavier A/A interfaces.

## 2. Materials and Methods

All Cu*_x_*(TiZrNb)_100−*x*_ NLGs were obtained by co-sputtering Cu (99.99%) and TiZrNb (99.9%) targets on Si (100) substrates, which was achieved using a PTL6S PVD system at room temperature. Two constituent phases of the Cu*_x_*(TiZrNb)_100−*x*_ NLGs are Cu_10_(TiZrNb)_90_ and Cu_40_(TiZrNb)_60_, respectively. These two phases have equal *h*, and the total thickness of all samples is 960 nm. The first layer of all samples is Cu_40_(TiZrNb)_60_, and the top layer is Cu_10_(TiZrNb)_90_. Before sputtering, the basic pressure inside the sputtering chamber was maintained below 6.5 × 10^−5^ Pa. During the sputtering process, Ar gas was supplied to stabilize the working pressure at 0.3 Pa. The two different components were realized by regulating the powers of the Cu target and TiZrNb target. The powers of the Cu target (TiZrNb target) used in the preparation of Cu_10_(TiZrNb)_90_ and Cu_40_(TiZrNb)_60_ components were 5 (150) and 30 (150) W, respectively, and the corresponding sputtering rates were 0.0110 (0.1649) and 0.0656 (0.1649) nm s^−1^, respectively. To attain the uniform thickness and microstructure of the samples, the rotation speed of the substrate was maintained at 30 rpm during the sputtering process. The individual layer thicknesses of the composites were selected in the range from several to tens of nanometers (5–80 nm) in order to investigate the size-dependent shear banding behaviors of the HEA-based nanolayered glass.

In order to analyze the Cu*_x_*(TiZrNb)_100−*x*_ NLGs, a series of characterizations were used, including X-ray diffraction (XRD, Bruker D8 Advance, Billerica, MA, USA), scanning electron microscopy (SEM, Tescan Mira 3, Brno, Czech Republic), and TEM (Talos F200X G2, Fulton, MD, USA) equipped with an energy dispersive spectroscopy (EDS) detector. TEM foils were prepared using a focused ion beam (FIB) system, specifically the FEI Helios Nanolab 600i. For evaluating the mechanical properties, a Nanoindenter system (Keysight, Agilent G200, Santa Clara, CA, USA) was employed to measure the hardness and shear instability of the Cu*_x_*(TiZrNb)_100−*x*_ NLGs. The measurements of the hardness and Young’s modulus were conducted under a continuous stiffness measurement mode based on the Oliver–Pharr method [57], with a total of 12 indentations acquired on each sample. The spacing between the indentations was 25 μm. The maximum depth of indentation was 400 nm, and a consistent strain rate of 0.05 s^−1^ was maintained in all indentation tests.

## 3. Results and Discussion

### 3.1. Microstructures of the Cu_x_(TiZrNb)_100−x_ NLGs

**Figure 1:**
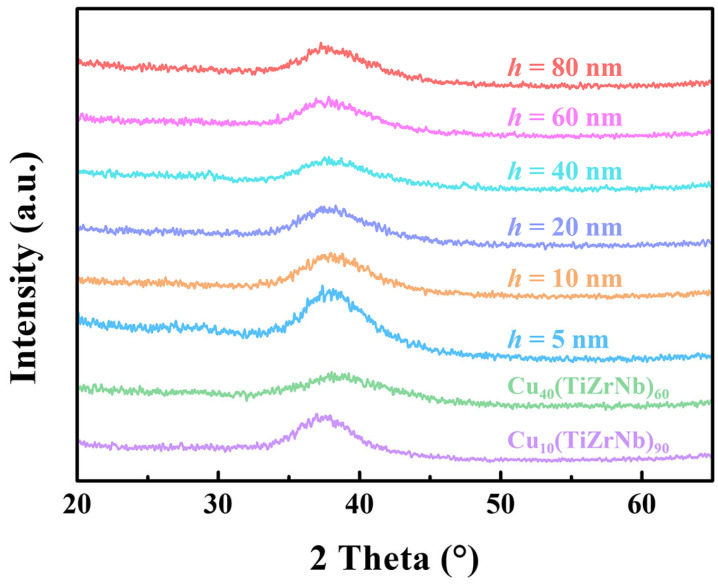
XRD patterns of the Cu_10_(TiZrNb)_90_ monolayer film, Cu_40_(TiZrNb)_60_ monolayer film, and Cu*_x_*(TiZrNb)_100−*x*_ NLGs with different layer thicknesses.

The structures of all the as-prepared samples were first analyzed by XRD. As shown in Figure 1, the XRD patterns of the Cu_10_(TiZrNb)_90_ monolayer film, Cu_40_(TiZrNb)_60_ monolayer film, and Cu*_x_*(TiZrNb)_100−*x*_ NLGs with *h* = 5–80 nm only present broad amorphous diffraction peaks without sharp crystal peaks, indicating that all samples are composed of amorphous structures.

**Figure 2:**
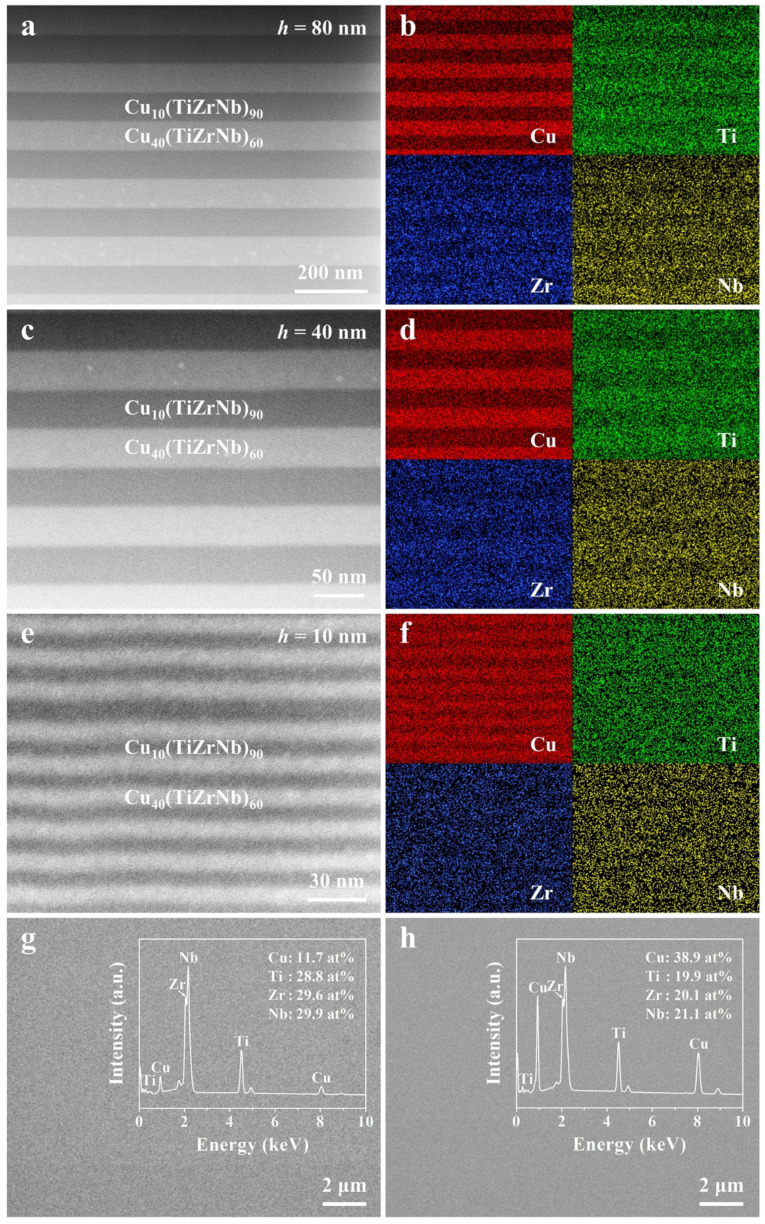
HAADF-STEM images and the corresponding EDS mapping images of the Cu*_x_*(TiZrNb)_100−*x*_ NLGs with *h* = (**a**,**b**) 80, (**c**,**d**) 40, and (**e**,**f**) 10 nm, respectively. SEM images and the corresponding EDS spectra of the (**g**) Cu_10_(TiZrNb)_90_ and (**h**) Cu_40_(TiZrNb)_60_ monolayer films, respectively.

High-angle annular dark-field scanning TEM (HAADF-STEM) and EDS were employed to investigate the internal structures and elemental distributions of three Cu*_x_*(TiZrNb)_100−*x*_ NLGs with *h* = 80, 40, and 10 nm. As displayed in Figure 2, it can be seen from the cross-sectional HAADF-STEM images that all samples have a layered modulation structure, and there is a conspicuous light and dark contrast between the two different phases. And all samples exhibit a perfect amorphous disordered structure, and no crystal particles are observed, which is well consistent with the above-mentioned XRD results. According to the HAADF-STEM measurements, the actual layer thicknesses of the Cu_10_(TiZrNb)_90_ and Cu_40_(TiZrNb)_60_ layers in the three Cu*_x_*(TiZrNb)_100−*x*_ NLGs are 78.7 ± 0.5 and 78.9 ± 0.3 nm at *h* = 80 nm, 38.9 ± 0.5 and 39.0 ± 0.3 nm at *h* = 40 nm, and 8.3 ± 0.6 and 8.4 ± 0.7 nm at *h* = 10 nm, respectively, which are well in accord with the designed layer thicknesses. In addition, it can be demonstrated from the EDS mapping images (Figure 2b,d,f) that various elements are all uniformly distributed in the three Cu*_x_*(TiZrNb)_100−*x*_ NLGs without any discernible element segregation, and all samples have a chemically alternating layer structure consisting of alternately stacked Cu-rich and Cu-poor layers. Moreover, in order to further verify the actual composition of the two phases, the Cu_10_(TiZrNb)_90_ and Cu_40_(TiZrNb)_60_ monolayer films were tested by EDS. The results (Figure 2g,h) manifest that the atomic percentages of Cu in the two phases are 11.7 at% and 38.9 at%, respectively, and the atomic percentages of Ti, Zr, and Nb in both phases are close to 1:1:1, which is within the error range of the design values.

**Figure 3:**
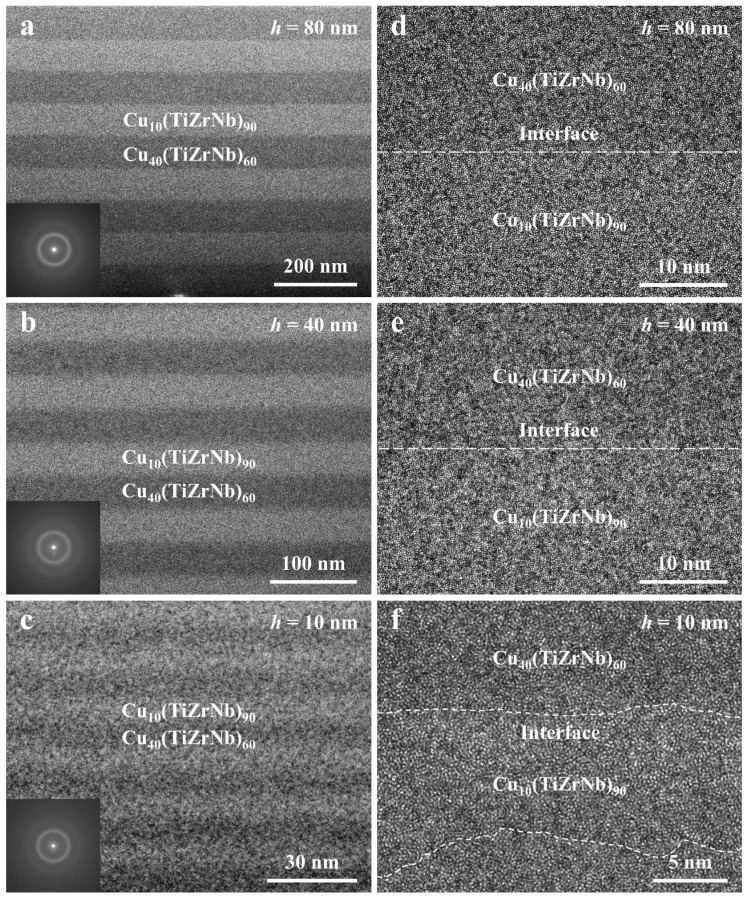
(**a**–**c**) The representative cross-sectional TEM images and (**d**–**f**) the corresponding HRTEM images of the Cu*_x_*(TiZrNb)_100−*x*_ NLGs with *h* = 80, 40, and 10 nm, respectively. Insets in (**a**–**c**) are the corresponding SAED patterns for the Cu*_x_*(TiZrNb)_100−*x*_ NLGs. The white dashed lines in (**d**–**f**) represent the interfaces between two phases.

In order to further characterize the details of the internal microstructures of the Cu*_x_*(TiZrNb)_100−*x*_ NLGs, TEM, selected area electron diffraction (SAED), and high-resolution TEM (HRTEM) were applied. As depicted by Figure 3a–c and their insets, all three samples exhibit good amorphous structures, and the SAED patterns of the three samples only show typical amorphous rings without diffraction spots assigned to the crystal structure. Moreover, when *h* = 80 and 40 nm, the interlayer interfaces of the samples are almost straight (Figure 3d,e). But, as *h* decreases to 10 nm, wavy interlayer interfaces are observed (Figure 3f). This phenomenon of the interlayer interfaces gradually becoming wavier as the layer thickness decreases is similar to that of the reported Ni_60_Nb_40_/Zr_50.7_Cu_28_Ni_9_Al_12.3_ NLGs [55].

### 3.2. Mechanical Behaviors of the Cu_x_(TiZrNb)_100−x_ NLGs

#### 3.2.1. The Hardness of the Cu_x_(TiZrNb)_100−x_ NLGs

**Figure 4:**
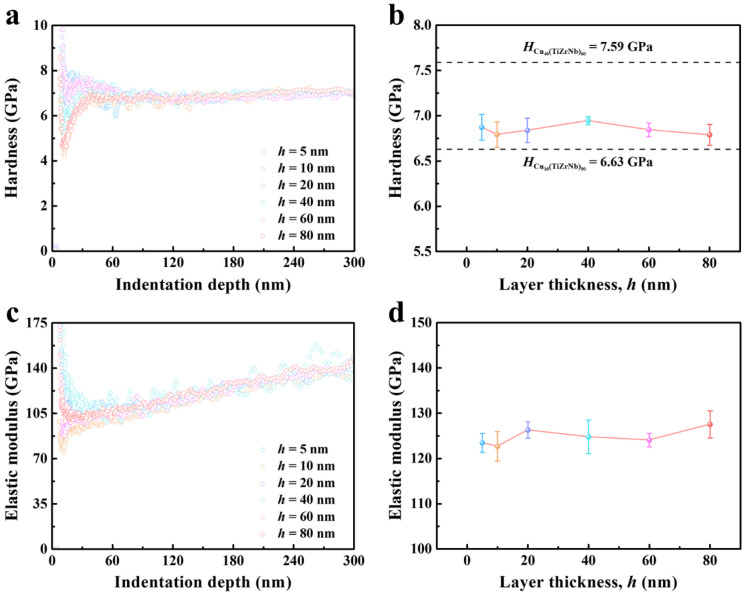
(**a**) The typical hardness–indentation depth curves and (**b**) the dependence of hardness on the layer thickness of the Cu*_x_*(TiZrNb)_100−*x*_ NLGs. (**c**) The typical elastic modulus–indentation depth curves and (**d**) the dependence of elastic modulus on the layer thickness of the Cu*_x_*(TiZrNb)_100−*x*_ NLGs.

The intrinsic hardness and elastic modulus of the Cu*_x_*(TiZrNb)_100−*x*_ NLGs with different layer thicknesses as well as the Cu_10_(TiZrNb)_90_ and Cu_40_(TiZrNb)_60_ monolayer films were tested through nanoindentation experiments. From the hardness–indention depth curves (Figure 4a) of the Cu*_x_*(TiZrNb)_100−*x*_ NLGs, it can be seen that the hardness values of all samples first increase rapidly and then decrease with the increase of indentation depth. This can be attributed to the fact that the radius of the indenter and the surface roughness of the samples affect the hardness values at extremely shallow indentation depth [58,59]. When the indentation depth increases to the range of 100–180 nm, the hardness values plateau. Afterwards, due to the substrate effect, the hardness values continue to increase with the increase of indentation depth. Therefore, the corresponding plateau hardness values at indentation depths of 10–15% of the total thickness of the samples are regarded as the intrinsic hardness values of the samples. As shown in Figure 4b, it can be observed that the hardness of the Cu*_x_*(TiZrNb)_100−*x*_ NLGs is not sensitive to changes in the layer thickness. Specifically, the hardness values of six samples with different layer thicknesses fluctuate within the range of 6.79–6.95 GPa. In addition, the hardness values of the six samples are higher than the hardness value of the Cu_10_(TiZrNb)_90_ monolayer film but lower than the hardness value of the Cu_40_(TiZrNb)_60_ monolayer film. Based on the rule of mixture (ROM), the predicted hardness value of the Cu*_x_*(TiZrNb)_100−*x*_ NLGs is obtained as follows:

(1) $$H_{\mathrm{ROM}}=f_{{\mathrm{Cu}}_{10}{\left(\mathrm{TiZrNb}\right)}_{90}}H_{{\mathrm{Cu}}_{10}{\left(\mathrm{TiZrNb}\right)}_{90}}+f_{{\mathrm{Cu}}_{40}{\left(\mathrm{TiZrNb}\right)}_{60}}H_{{\mathrm{Cu}}_{40}{\left(\mathrm{TiZrNb}\right)}_{60}}$$

where $f_{{\mathrm{Cu}}_{10}{\left(\mathrm{TiZrNb}\right)}_{90}}$ and $f_{{\mathrm{Cu}}_{40}{\left(\mathrm{TiZrNb}\right)}_{60}}$ are the volume fractions of the two phases, respectively, equal to 0.5, $H_{{\mathrm{Cu}}_{10}{\left(\mathrm{TiZrNb}\right)}_{90}}$ = 6.63 GPa, and $H_{{\mathrm{Cu}}_{40}{\left(\mathrm{TiZrNb}\right)}_{60}}$ = 7.59 GPa. The calculation result indicates that *H*_ROM_ = 7.11 GPa, which is higher than the actual hardness values of the six Cu*_x_*(TiZrNb)_100−*x*_ samples. It is demonstrated from the above finding that the strengthening effect of the layered structure cannot be reflected in the Cu*_x_*(TiZrNb)_100−*x*_ NLGs. Similar to the fact that the hardness values do not change significantly, the elastic modulus values fluctuate in the interval of 122.67–127.52 GPa when *h* changes in the range of 5–80 nm (Figure 4c,d).

#### 3.2.2. The Shear Instability of the Cu_x_(TiZrNb)_100−x_ NLGs

**Figure 5:**
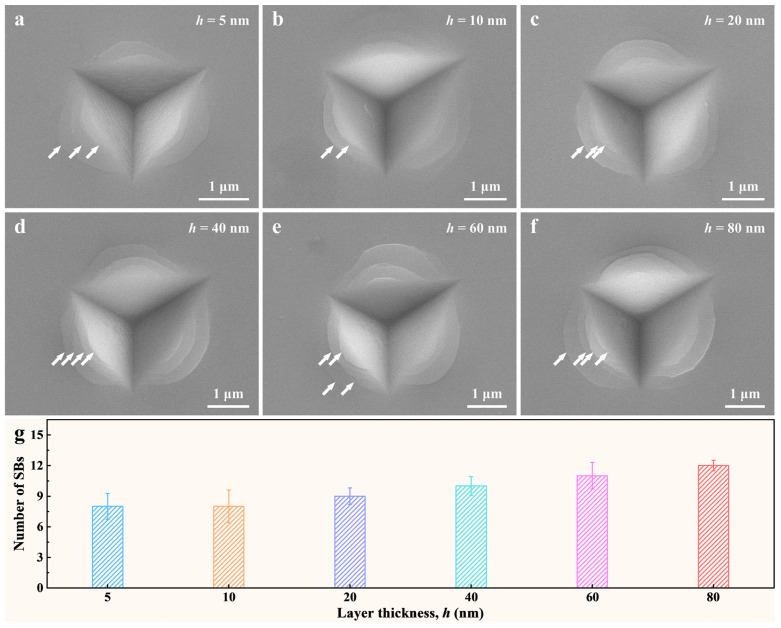
(**a**–**f**) SEM images of the indentations of the Cu*_x_*(TiZrNb)_100−*x*_ NLGs with *h* = 5–80 nm. The white arrows point to well-shaped circular SBs. (**g**) The number of SBs on the indentation surfaces of the Cu*_x_*(TiZrNb)_100−*x*_ NLGs with different layer thicknesses.

Following the nanoindentation experiments, SEM was utilized to investigate the residual indentation morphologies of the Cu*_x_*(TiZrNb)_100−*x*_ NLGs with various layer thicknesses. From Figure 5a–f, it can be seen that at an indention depth of 400 nm, well-shaped circular SBs appear on the indentation surfaces for all samples with *h* = 5–80 nm, as indicated by the white arrows. By analyzing the number of SBs, it is found that there is a strong dependence between the number of SBs and the layer thickness. That is, the number of SBs increases monotonically with the increase of layer thickness. Specifically, as shown in Figure 5g, the number of SBs on the indentation surfaces of the six Cu*_x_*(TiZrNb)_100−*x*_ NLGs increases from 8 at *h* = 5 nm to 12 at *h* = 80 nm, implying that the shear instability increases monotonically with the increase of layer thickness.

**Figure 6:**
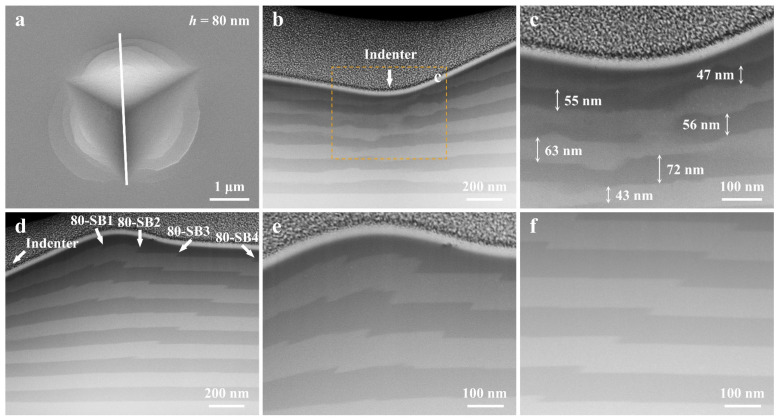
(**a**) SEM image and (**b**–**f**) the corresponding cross-sectional STEM images of the indentation of the Cu*_x_*(TiZrNb)_100−*x*_ NLG with *h* = 80 nm. (**b**,**c**) show the deformation behavior underneath the indenter while (**d**–**f**) present the internal shear banding behavior, in which (**c**) is derived from the boxed region in (**b**) and (**e**,**f**) are amplified images from (**d**). The white solid line in (**a**) designates where the indentation was cross-sectioned. The positions of the indenter and the SBs are indicated by white arrows in (**b**,**d**).

In order to further analyze the deformation behaviors of the areas underneath the indenter and the SB areas near the indenter, the post-mortem TEM measurements were performed on the Cu*_x_*(TiZrNb)_100−*x*_ NLGs with *h* = 80, 40, and 10 nm. Figure 6a presents the SEM image of the nanoindentation surface of the sample with *h* = 80 nm, and the white solid line represents the cross-sectional position used for preparing TEM lamellas by the FIB technique. For the Cu*_x_*(TiZrNb)_100−*x*_ NLG with *h* = 80 nm, the obvious inhomogeneous thinning of the constituent layers is observed in the area underneath the indenter (Figure 6b,c). Consistent with the number of SBs on the nanoindentation surface observed by the SEM image, four SBs in the region next to the indenter are also revealed by the corresponding cross-sectional STEM image of the Cu*_x_*(TiZrNb)_100−*x*_ NLG with *h* = 80 nm (Figure 6d). According to the distance between the SBs and the indenter, from near to far, the four SBs are named 80-SB1, 80-SB2, 80-SB3, and 80-SB4, respectively. The interlayer interfaces within the SBs underwent severe shear, forming sharp angles with the original horizontal direction (Figure 6e,f). This type of SB is defined as the cutting-like SB. Obviously, the four SBs are all cutting-like SBs that do not penetrate all constituent layers. The reason for the difference in deformation behaviors between the area underneath the indenter and the SB area next to the indenter is that the stress states in the two areas are different. Under ideal conditions, the area underneath the indenter is only subject to normal stress, which is consistent with the stress state in the micropillar compression test. In addition to the normal stress, there is also shear stress in the area next to the indenter, which is a complex stress state.

**Figure 7:**
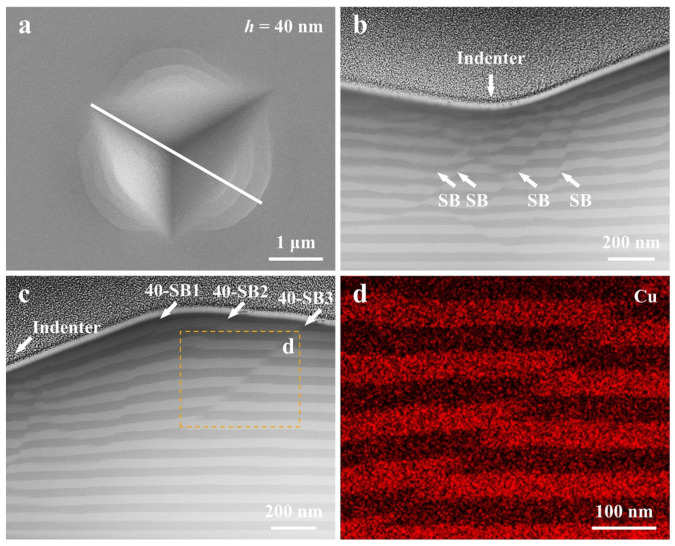
(**a**) SEM image, the corresponding cross-sectional (**b**,**c**) STEM images, and (**d**) EDS mapping image of the indentation of the Cu*_x_*(TiZrNb)_100−*x*_ NLG with *h* = 40 nm. The white solid line in (**a**) represents where the indentation was cross-sectioned. (**d**) is derived from the boxed region in (**c**). The positions of the indenter and the SBs are indicated by white arrows in (**b**,**c**).

Similar to the results at *h* = 80 nm, the Cu*_x_*(TiZrNb)_100−*x*_ NLG with *h* = 40 nm exhibits several well-shaped circular SBs on the nanoindentation surface (Figure 7a). But unlike from the sample with *h* = 80 nm, the Cu*_x_*(TiZrNb)_100−*x*_ NLG with *h* = 40 nm exhibits multiple SBs in the area underneath the indenter to accommodate the plastic deformation, as shown by the white arrows in Figure 7b. In addition, three SBs are identified in the area near the indenter (Figure 7c), and the number of SBs is consistent with that observed on the nanoindentation surface. According to the distance between the SBs and the indenter, from near to far, the three SBs are defined as 40-SB1, 40-SB2, and 40-SB3, respectively. Notably, the three SBs only penetrate a portion of the constituent layers and do not penetrate the entire sample. Unlike the case of the sample with *h* = 80 nm, the SBs formed in the sample with *h* = 40 nm in the area near the indenter are not all cutting-like SBs. Unlike the cutting-like SB formed by severe shear, the kinking-like SB is formed by the cooperative kinking of the constituent layers along the shear direction. Evidently, the 40-SB1 and 40-SB2 are a combination of the kinking-like and cutting-like SBs. The bottom of these two SBs displays the kink of the constituent layers, while severe shear occurs at the top of these two SBs. The 40-SB3 in all constituent layers are unified cutting-like SBs, as shown in the enlarged EDS image in Figure 7d. In summary, the Cu*_x_*(TiZrNb)_100−*x*_ NLG with *h* = 40 nm exhibits different deformation modes in the areas underneath and adjacent to the indenter compared to the sample with *h* = 80 nm.

**Figure 8:**
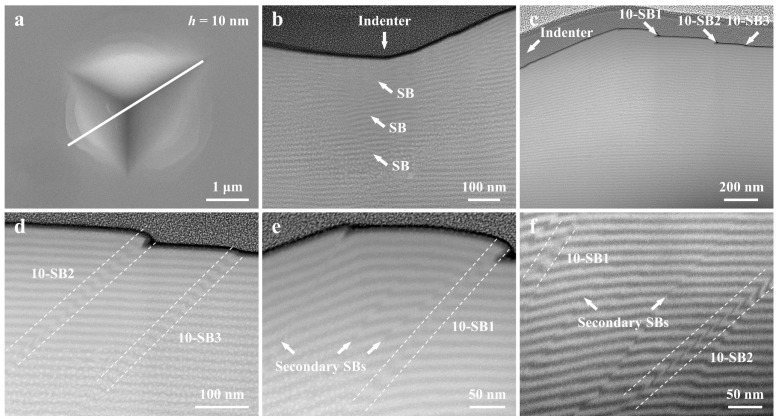
(**a**) SEM image, and (**b**–**f**) the corresponding cross-sectional STEM images of the indentation of the Cu*_x_*(TiZrNb)_100−*x*_ NLG with *h* = 10 nm. The white solid line in (**a**) represents where the indentation was cross-sectioned. The positions of the indenter are indicated by white arrows in (**b**,**c**). The positions of SBs are indicated by white arrows and white dashed lines in (**b**–**f**).

The Cu*_x_*(TiZrNb)_100−*x*_ NLG with *h* = 10 nm exhibits a relatively small number of SBs on the nanoindentation surface (Figure 8a). Similar to the case of the sample with *h* = 40 nm, the plastic deformation of the Cu*_x_*(TiZrNb)_100−*x*_ NLG with *h* = 10 nm is also accommodated by multiple SBs in the area underneath the indenter (Figure 8b). Moreover, three primary SBs can be observed in the area near the indenter, as indicated by the white arrows in Figure 8c, which is consistent with the number of SBs observed on the indentation surface. According to the distance between the SBs and the indenter, from near to far, the three primary SBs are named 10-SB1, 10-SB2, and 10-SB3, respectively. These three primary SBs are typical kinking-like SBs (Figure 8d), and none of them penetrate the entire sample. Significantly different from the first two samples, in addition to the primary SBs, many secondary SBs are also observed in the sample with *h* = 10 nm, as indicated by the arrows in Figure 8e,f. The formation of secondary SBs is due to the fact that the formation of primary SBs is not enough to eliminate the stress of the surrounding matrix, and other locations still meet the yield conditions [8]. Therefore, the plastic deformation is accommodated by these secondary SBs together with primary SBs.

The above results demonstrate that the deformation behaviors of the Cu*_x_*(TiZrNb)_100−*x*_ NLGs in the areas underneath and near the indenter is highly correlated with the layer thickness. In the area underneath the indenter, the deformation behaviors of the Cu*_x_*(TiZrNb)_100−*x*_ NLGs change from the non-uniform thinning of the constituent layers at *h* = 80 nm to multiple shear bandings at *h* = 40 and 10 nm as *h* decreases. Existing tensile and compression experiments have corroborated that the multiple shear banding behavior can make the deformation of MGs more uniform under uniaxial stress [48,60]. In the region near the indenter, the deformation behavior of the Cu*_x_*(TiZrNb)_100−*x*_ NLGs changes from four cutting-like SBs at *h* = 80 nm to three SBs at *h* = 40 nm. And only the 40-SB3 is a complete cutting-like SB, and the other two SBs exhibit a combination of the kinking-like and cutting-like SBs. When *h* further decreases to 10 nm, three kinking-like primary SBs appeared, accompanied by multiple secondary SBs. A large number of nano/microindentation experiments have validated that the reduction in the number of SBs corresponds to the reduction in the shear instability of the sample under a complex stress state [21,43,55,61,62]. In addition, the shear instability of the cutting-like SBs is generally greater than that of the kinking-like SBs [21,61]. Thus, the shear instability resistance of the Cu*_x_*(TiZrNb)_100−*x*_ NLGs gradually increases with the decrease of layer thickness. However, further precise quantitative analysis about the samples under three layer thicknesses is still needed to draw this conclusion.

**Figure 9:**
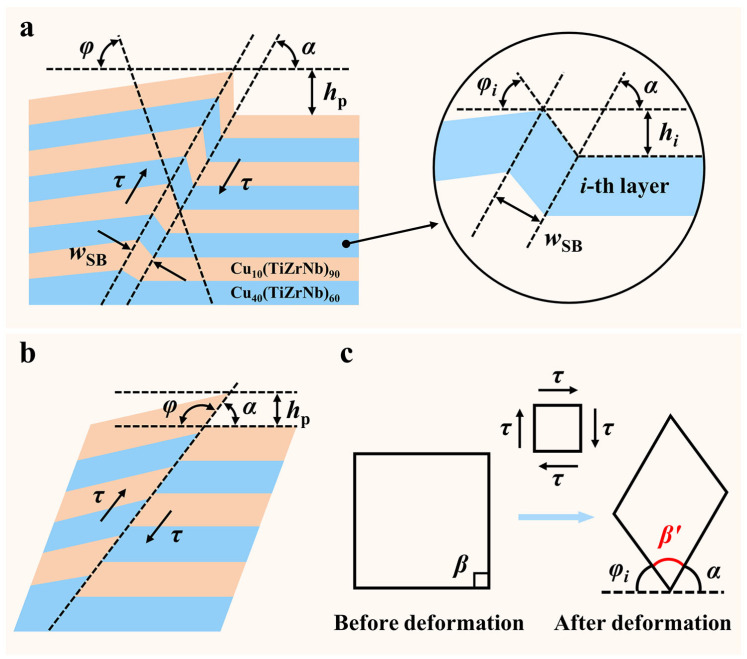
Schematic diagrams of the physical models of the (**a**) kinking-like and (**b**) cutting-like SBs, and (**c**) the corresponding microelement deformation within the given SB under a pure shear stress state in the Cu*_x_*(TiZrNb)_100−*x*_ NLGs. In the above schematic diagram, *β* and *β*′ are the angles before and after microelement deformation, respectively. The magnified image in (**a**) shows the geometric model details of the *i*-th layer, where *h_i_* and *φ_i_* are shear displacement and interface kink angle of the *i*-th layer within the SB, respectively.

In order to accurately quantify the degrees of shear instability within the SBs of the samples with different layer thicknesses, a theoretical model including two SB modes, i.e., the kinking-like and cutting-like SBs, was proposed based on the previous work of our group [21] (Figure 9). It was assumed that the SB region is subject to a pure shear stress state. According to the characteristics of the kinking-like and cutting-like SBs, they are simplified into geometric models, as shown in Figure 9a,b. Based on the microelement within the SB shown in Figure 9c, the shear strain (*γ*) is calculated as follows:

(2) $$\gamma_{i}=\beta -\beta^{\prime }=\varphi_{i}+\alpha -\frac{\pi }{2}$$

where *i* represents the *i*-th layer from bottom to top, *α* is the angle between the SB and the original interlayer interface, and *φ* is the kink angle between the interlayer interface within the SB and the original interlayer interface. Because *φ* is in the range of [0, *π* − *α*], the maximum value of *γ* is π/2 according to Formula (2). Moreover, since *φ* and *α* of the cutting-like SB are almost complementary (Figure 9b), the *γ* value of the cutting-like SB is close to the maximum value. In addition to *γ*, a new shear localization parameter (*ξ*_p_) is also introduced in the model to characterize the degree of the shear localization of the SB. The calculation method is as follows:

(3) $$\xi_{p}=\frac{h_{p}}{w_{\mathrm{SB}}}$$

where *h*_p_ and *w*_SB_ are the pile-up height induced by shear banding and the width of the SB, respectively.

**Figure 10:**
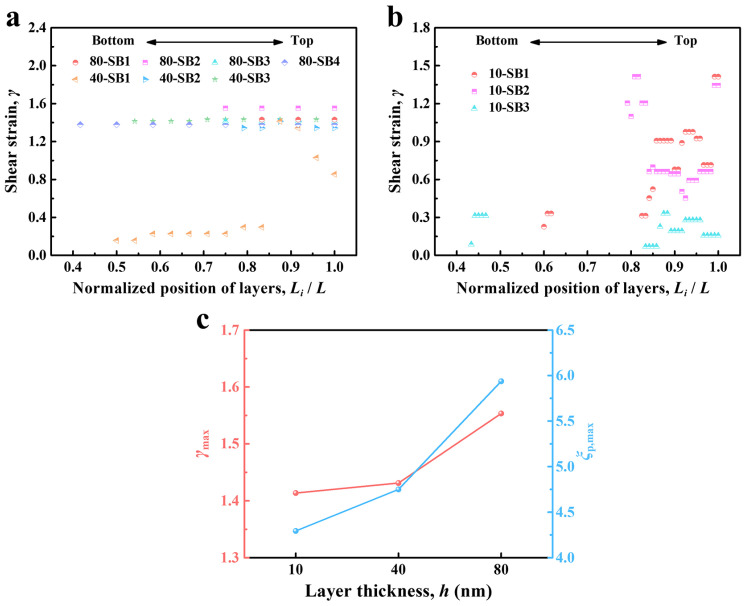
(**a**,**b**) The calculated *γ* within different SBs and (**c**) the corresponding calculated *γ*_max_ and *ξ*_p,max_ of the Cu*_x_*(TiZrNb)_100−*x*_ NLGs with *h* = 80, 40, and 10 nm, respectively. *L_i_*/*L* denotes the normalized position of the constituent layers in each sample, where *L_i_* is the position of the *i*-th layer from bottom to top and *L* is the total thickness of each sample.

Based on the models shown in Figure 9, the relevant parameters of the SBs of the Cu*_x_*(TiZrNb)_100−*x*_ NLGs with *h* = 80, 40, and 10 nm were measured, and *α*, *w*_SB_, and *h*_p_ are listed in Table 1. Of note, the SBs measured here are limited to those named previously, excluding multiple SBs in the area underneath the indenter and several secondary SBs. From Table 1, it can be seen that the *α* values of the SBs generated under these three layer thicknesses are all around 45°, which proves that the pure shear stress state assumed by the models is reasonable. Based on the measured *φ* of each layer, the shear strain values of each layer of the Cu*_x_*(TiZrNb)_100−*x*_ NLGs with *h* = 80, 40, and 10 nm were calculated. As shown in Figure 10a, for the cutting-like SBs (such as 80-SB1, 80-SB2, 80-SB3, and 80-SB4), the shear strain values of different layers are almost the same, that is, the *φ* values within the cutting-like SBs are almost constant. Furthermore, it can be seen through careful comparison that the shear strain of the sample with *h* = 80 nm is overall higher than that of the sample with *h* = 40 nm, and the shear strain of the sample with *h* = 10 nm is much lower than that of the first two samples (Figure 10b). The maximum shear strain value (*γ*_max_) in each sample is selected as the criterion for judging the degree of shear instability of the sample. As shown in Figure 10c, the *γ*_max_ values of the Cu*_x_*(TiZrNb)_100−*x*_ NLGs with *h* = 80, 40, and 10 nm are 1.55, 1.43, and 1.41, respectively, indicating that the degrees of shear instability of the Cu*_x_*(TiZrNb)_100−*x*_ NLGs monotonically decrease with the decrease of *h*. The maximum values of strain localization parameter (*ξ*_p,max_) for the Cu*_x_*(TiZrNb)_100−*x*_ NLGs with *h* = 80, 40, and 10 nm are 5.94, 4.75, and 4.29, respectively, implying that the degrees of shear instability are reduced by 28% when *h* decreases from 80 to 10 nm. Beyond that, the calculated *ξ*_p,max_ values monotonically decrease with the decrease of *h*, which is consistent with the above-mentioned *γ*_max_ – *h* relationship. The above two parameters (i.e., *γ*_max_ and *ξ*_p,max_) jointly prove that the reduction of layer thickness improves the resistance to the shear instability of the Cu*_x_*(TiZrNb)_100−*x*_ NLGs.

**Figure 11:**
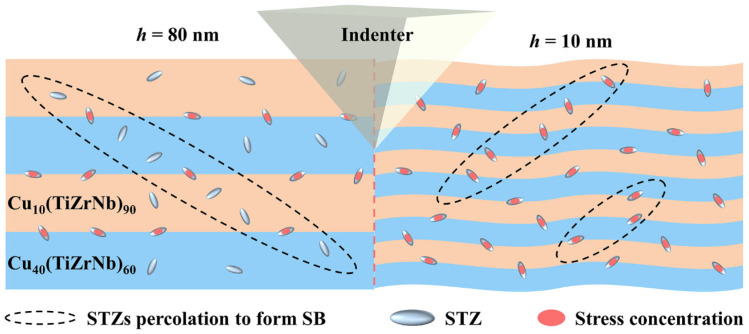
Schematic diagram of deformation mechanisms of the Cu*_x_*(TiZrNb)_100−*x*_ NLGs with *h* = 80 and 10 nm under the nanoindentation experiments.

As the layer thickness decreases, the reason for the transformation of the deformation modes and the enhancement of the resistance to shear instability is the increase in quantity and the change in the morphology of the A/A interfaces. As shown in Figure 11, stress concentration preferentially occurs at the A/A interfaces due to the high free volume of the A/A interfaces [63,64]. When the stress concentration reaches a certain level, STZs are activated [50,65]. These STZs will continue to diffuse along the direction of maximum shear stress within the constituent layers until reaching the next A/A interfaces. Due to the large layer thickness (*h* = 80 nm), the STZs need to penetrate fewer interfaces. Moreover, the A/A interfaces are straight, jointly making it easy for the STZs to penetrate the A/A interfaces and reach a critical size for generating mature SBs. Therefore, multiple cutting-like SBs appear in the region near the indenter for the Cu*_x_*(TiZrNb)_100−*x*_ NLG with *h* = 80 nm.

When *h* = 10 nm, the number of A/A interfaces increases significantly, and a large number of STZs appear in the interface regions. However, the formation of mature SBs needs to penetrate many interfaces, which greatly increases the resistance to the propagation of the SBs [55]. When the penetration of STZs is obstructed, they may first deviate along the direction of A/A interfaces, and then penetrate in some relatively weak areas [66]. In this case, the mature SBs can also be formed in NLGs with smaller layer thickness. However, the interfaces of the Cu*_x_*(TiZrNb)_100−*x*_ NLG with *h* = 10 nm are wavy, which may hinder the deflection of STZs and thus inhibit the formation of mature SBs. Therefore, in addition to the kinking-like primary SBs, a large number of secondary SBs appear in the area near the indenter to jointly accommodate the plastic deformation when *h* =10 nm.

## 4. Conclusions

In this work, the Cu*_x_*(TiZrNb)_100−*x*_ NLGs with different layer thicknesses were prepared by magnetron sputtering, and the layer thickness dependence of the deformation behaviors in the areas underneath and near the indenter in the nanoindentation experiments was systematically studied. Also, the degrees of shear instability of the samples with different layer thicknesses were accurately quantified through a theoretical model. The main conclusions are as follows:

(1) The hardness and elastic modulus of the Cu*_x_*(TiZrNb)_100−*x*_ NLGs are not sensitive to changes in the layer thickness. Specifically, in the range of *h* = 5–80 nm, the values of hardness and elastic modulus of the six samples fluctuate within the ranges of 6.79–6.95 GPa and 122.67–127.52 GPa, respectively.
(2) The deformation behaviors of the Cu*_x_*(TiZrNb)_100−*x*_ NLGs in the areas underneath the indenter change with the decrease of layer thickness. In the areas underneath the indenter, the sample with *h* = 80 nm is deformed by the uneven thinning of the constituent layers, while the deformation of the samples with *h* = 40 and 10 nm is accommodated by multiple SBs.
(3) In the areas near the indenter, the formed SBs are all cutting-like SBs for the Cu*_x_*(TiZrNb)_100−*x*_ NLG with *h* = 80 nm, a combination of the cutting-like and kinking-like SBs is observed for the sample with *h* = 40 nm, and the kinking-like primary SBs and many secondary SBs are appeared in the sample with *h* = 10 nm.
(4) The resistance to shear instability of the Cu*_x_*(TiZrNb)_100−*x*_ NLGs increases monotonically with the decrease of layer thickness. When *h* decreases from 80 to 10 nm, the *ξ*_p,max_ values of the samples decrease from 5.94 to 4.29. The improvement in shear instability resistance comes from the increase in the number of A/A interfaces and the A/A interfaces becoming wavier, which can effectively block the propagation of SBs.

In summary, the present experimental and theoretical studies clearly show that the shear banding-induced strong strain localization of the amorphous alloys can be significantly alleviated by introducing nanolayered structures. The underlying origin is the transition of deformation modes from cutting-like to kinking-like shear banding due to the refining of the nanolayered structure down to 10 nm. The advantage of the design is the creation of the unique homogeneous A/A interfaces that are produced by a single HEA system. This study provides an easy and flexible route to develop ultra-strong yet highly deformable amorphous alloys that could be widely employed in practical engineering applications. In addition, the proposed theoretical model provides a simple and convenient way to evaluate the strain localization of amorphous alloys in combination with simple nanoindentation tests that are easy to access by researchers.

## Figures and Tables

**Table 1:** Summary of *α*, *w*_SB_, and *h*_p_ for the Cu*_x_*(TiZrNb)_100−*x*_ NLGs with *h* = 80, 40, and 10 nm.

SB	*α* (^o^)	*w*_SB_ (nm)	*h*_p_ (nm)
80-SB1	58	21	101
80-SB2	51	16	95
80-SB3	46	13	51
80-SB4	41	10	21
40-SB1	67	13	49
40-SB2	47	18	67
40-SB3	40	8	38
10-SB1	48	17	73
10-SB2	44	12	30
10-SB3	46	9	8

## Data Availability

Data are contained within the article.
